# Implementation and Experimental Application of Industrial IoT Architecture Using Automation and IoT Hardware/Software

**DOI:** 10.3390/s24248074

**Published:** 2024-12-18

**Authors:** David Calderón, Francisco Javier Folgado, Isaías González, Antonio José Calderón

**Affiliations:** Department of Electrical Engineering, Electronics and Automation, University of Extremadura, Avenida de Elvas, s/n, 06006 Badajoz, Spain; davidcg@unex.es (D.C.); ffolgar@unex.es (F.J.F.); ajcalde@unex.es (A.J.C.)

**Keywords:** IIoT, Industry 4.0, functional architecture, monitoring, Grafana, open-source, microgrid

## Abstract

The paradigms of Industry 4.0 and Industrial Internet of Things (IIoT) require functional architectures to deploy and organize hardware and software taking advantage of modern digital technologies in industrial systems. In this sense, a lot of the literature proposes and describes this type of architecture with a conceptual angle, without providing experimental validation or with scarce details about the involved equipment under real operation. Aiming at overcoming these limitations, this paper presents the experimental application of an IIoT architecture divided into four functional layers, namely, Sensing, Network, Middleware and Application layers. Automation and IoT hardware and software are used to implement and apply the architecture. Special attention is put on the software Grafana, chosen in the top layer to deploy graphical user interfaces that are remotely accessible via web. A pilot microgrid integrating photovoltaic energy and hydrogen served as scenario to test and prove the suitability of the architecture in four application cases.

## 1. Introduction

The Industrial Internet of Things (IIoT) is one of the most relevant enabling technologies of the Industry 4.0 (I4.0) [1] that consists of the application of the IoT in the industry. Namely, the IIoT is applied to connect machines and devices in industrial environments, focusing on machine-to-machine communication; any failure can lead to high-risk losses and the amount of data collected in IIoT is much larger than in IoT [2]. Furthermore, IIoT is also considered an I4.0 equivalent concept in some of the literature [3].

Nowadays, IIoT technologies are changing the traditional hierarchical structures and are providing more scalable systems where data can be exchanged in a seamless manner [4]. In this sense, hardware equipment, software and communications need to be organized and distributed following functional architectures, which attract research attention for the I4.0 and IIoT paradigms. For example, the Reference Architectural Model for Industrie 4.0 (RAMI 4.0) is a three-dimensional map proposed by the Platform Industrie 4.0 in 2015. In a similar sense, IoT-oriented architectures are divided into layers or levels that exchange information using IoT technologies.

Various recent publications review existing architectures and propose new ones [3,5,6,7,8,9]. However, there are two drawbacks to highlight in this regard. On the one hand, a paradox occurs because new models and frameworks are developed to overcome the limitations of previous architectures, giving place to a variety of proposals that become again a source of heterogeneity and misunderstanding [5]. On the other hand, the degree of complexity and abstraction of these architectures is a barrier for their real industry application [3,7,10].

In any case, the research on functional architectures is a trend in automation and supervision applied to I4.0 and IIoT-enabled factories [7,11]. For instance, a review of IIoT architectures and technologies is found in [6], placing the focus on security issues. Lombardi et al. [12] present an overview of IoT architectures, protocols and applications. Mirani et al. [7] perform a review of IIoT architectures and the key challenges. An interesting conclusion of the paper is that the focus on providing experiment-based architectures is increasing over time [7]. Another review about IIoT architectures implementation is found in [13], putting the focus on fog computing and Manufacturing Execution Systems (MES). That work states that implemented IIoT applications are scarce and that most of the architectures are hypothetical, studied using software simulations, virtual environments or use cases providing scarce implementation information, which are scenarios that are far from real applications for industrial processes [13].

In [14], an IIoT architecture is developed around the Open Platform Communications (OPC) protocol including details about the configuration of a Python middleware to handle OPC connections. A proposal based on seven layers is found in [15] without providing neither configuration details nor experimental validation. In [16], an architecture to integrate 5G and IIoT technologies is proposed, and a use case is described without experimental data. A conceptual architecture for I4.0 is introduced in [17] discussing its implications without providing any type of validation. A multi-layered IIoT platform is studied in a real-world testbed in [18], putting the focus on telecommunications aspects. In [19], an IoT architecture is implemented by means of a modular IoT system for indoor quality monitoring, the effectiveness of which is tested by comparing data from 84 homes of families with children.

In other publications, there is experimental validation at a laboratory-scale and/or using small-scale equipment. For instance, in [20], an open IIoT platform is presented and validated in two experimental prototypes involving robotic tasks. An application-oriented architecture is presented in [21] involving IoT hardware and software, providing a snapshot of a monitoring interface as experimental data. A three-tiered architecture for IIoT is proposed in [22], being validated in an experimental benchmark through a visualization dashboard. A microservices architecture for machine monitoring in the IIoT is presented in [23] and validated in a testbed, putting the focus on computation and communication aspects. Oriented towards buildings maintenance, a four-layered IoT architecture is studied at a laboratory scale in [24]. Ref. [25] proposes system architecture to integrate MES in IIoT and to partly implement and evaluate it in three laboratory scale cases. Another case is found in [26], where an architecture for smart factories fully based on open-source software is proposed and validated in a small-scale pilot testing. A so-called intelligent automation pyramid is presented in [27] and validated in a small-scale testbed including a PLC, a robotic arm and LabVIEW software. Furthermore, some architectures are validated using simulation like in the following works. An IIoT architecture oriented towards cybersecurity is presented in [28], evaluated using simulated data. A secured IIoT architecture is proposed in [1] and studied based on a TON_IoT dataset. An IIoT framework for monitoring crude oil production is presented in [29], being validated through a simulation case study.

It must be remarked that theoretical and conceptual proposals are valuable contributions, but there is a pressing need of reporting experimental and real-world validation of IIoT architectures to foster its deployment. The lack of details and the conceptual nature of most of architectures proposed in the literature are serious drawbacks for the applicability of these architectures [7,10,11].

Many of these functional architectures are based on open-source technology. In fact, the usage of open-source hardware and software in I4.0 and IIoT is an increasing trend as witnessed in the recent scientific literature [14,15,20,26,30,31,32,33,34,35]. Open-source projects are key accelerators for the industry adoption of IoT [36]. Proposals related to I4.0, IIoT and smart factories using open-source software represent an open opportunity for research and industrial application [26]. Moreover, IoT open-source tools also contribute to handling interoperability [31,37], mainly in the industrial context, where a variety of hardware and software nodes must be connected to share data [10,38]. Open-source solutions provide the ability of modifying the source code at a low level and new developments can be tested without relevant economic risks [30,39]. This way, enterprises normally have very specific use-case-tailored solutions that need to be developed and tested, and commercial solutions often lack the required level of adaptability [40]. A ready-made industrial-grade IIoT solution with high levels of robustness and reliability can be provided by automation manufacturers such as Bosch, ABB, Siemens, etc. [40]. However, expensive implementation costs, proprietary software and vendor “lock-in” are well-known disadvantages [40]. In contrast, low-cost and IoT open-source technologies help to reduce the required budget and solve the financial burden [26] as well as to provide implementation simplicity thanks to open libraries and standards [40].

On the other hand, industrial monitoring and automation can be implemented using traditional solutions involving equipment like Programmable Logic Controllers (PLCs) and Supervisory Control and Data Acquisition (SCADA) systems from well-established manufacturers. The PLC has been the key building block of industrial control systems throughout the whole automation revolution [41]. PLCs are still present in existing factories, even with many years of remaining useful life. Moreover, new industrial production facilities are being equipped with such equipment due to their proven reliability when managing critical assets and tasks. In fact, PLCs are considered an essential part within the I4.0 and IIoT approaches [42].

Consequently, functional architectures solely based on IoT open-source hardware/software technology are not applicable to many industries, and higher degrees of maturity is still required. In this regard, an architecture that combines both automation equipment and IoT technologies can be suitable to organize the heterogeneous hardware, software and communications imposed in real-practice industrial systems.

To this purpose, in this paper, the implementation and application of an IIoT architecture in a set of experimental cases is presented. Different industrial automation and IoT open-source equipment (hardware, software and communication means) are used and integrated following the architecture in order to prove its feasibility and suitability under real operating conditions. It must be noted that this research does not present a new IIoT architecture but validates the well-known four-layered architecture in a real-world facility.

The software package chosen for the experimental validation of the architecture was Grafana. This is an open-source software package profusely used in the IoT context for data visualization through web-based interfaces [34,43,44]. Grafana is placed in the top layer, the so-called Application layer where it plays the role of visualization and monitoring systems with successful results. In some papers, certain attention is put on the developed Grafana interface, even highlighting some features or functionalities such as user-friendly and responsive visualization [21,34,45], easy customization of graphical representations [33], clear dashboard visuals [46], ability to display data in a variety of formats [47] or available data selection options (time range, presentation interval, zooming, etc.) [34,48,49]. However, in most cases, this software is scarcely mentioned despite its relevance for monitoring purposes. For example, in [23,40,43,44,50,51,52,53,54,55] there are no explanations about the software, configuration or user interfaces. Therefore, given the increasing application of Grafana software in advanced facilities, it deserves a paper to provide a view of its main features focused on industrial systems emphasizing its suitability for I4.0 and IIoT paradigms.

Regarding the experimental validation, among the most common application scenarios of the IIoT, energy production is found [4]. In this sense, a pilot microgrid based on renewable energy hybridized with hydrogen is used as a real-world application scenario. Such a microgrid involves distributed energy production, storage and consumption with the corresponding equipment for sensing, control and monitoring, being a representative facility for the purpose of the present work. This way, four experimental cases are reported to demonstrate the suitability of the architecture to monitor and manage data in IIoT. The main contributions of this work are listed as follows:

- Open IoT technologies (hardware, software and communications) are integrated with traditional automation equipment.
- Data are acquired, stored and visualized in a seamless manner, putting a special focus on the monitoring software environment Grafana.
- Networked remote access is allowed through a custom-tailored web user interface.
- In contrast to conceptual and/or simulation frameworks, experimental results under real operating conditions prove the validity of the architecture.

The structure of the rest of the manuscript is as follows: after the Section 1, the Section 2 provides a brief overview about Grafana fundamentals and application cases in the recent literature. The Section 3 overviews functional architectures in an industrial context and describes the architecture applied in this research. In the Section 4, the suitability and validity of the proposal is proven through a set of experimental cases developed around a microgrid. Finally, the main conclusions of the work are addressed.

## 2. Fundamentals of Grafana and a Brief Review of Grafana Applications

### 2.1. Grafana Main Features

Grafana was created in 2013 and was conceived as an open-source tool for easy visualization and querying data from any source [56]. Nowadays, Grafana is an IoT open-source, multi-platform and lightweight software suite with built-in connectivity with a large number of databases. In this sense, examples of compatible databases are MariaDB, MySQL, Microsoft SQL, InfluxDB, PostgreSQL, Prometheus, etc. In addition, data extracted from different databases can be displayed together, even as such databases are hosted in different locations (in-house or cloud-hosted). The “plug and play” nature of Grafana allows multiple database sources to be “plugged” in for easy querying [47].

As asserted by Ayele et al. [35], an open research issue in IIoT is the customization and design of user-friendly interfaces. In this regard, Grafana provides a web-based interface composed of a general-purpose dashboard and a graph composer to develop Graphical User Interfaces (GUI) in an accessible and versatile manner. It facilitates the design of highly customizable GUI, mainly displaying time-series charts and instant values with a responsive interaction.

From the point of view of the server, Grafana is multi-platform and lightweight, so the computing device that plays the role of server is not restricted to a particular operative system and does not need high computational resources. For example, MS Windows, OS and Linux-based computers can be used as Grafana servers. Furthermore, Single Board Computers (SBC) such as Raspberry Pi can host the server. Concerning the installable package, currently, there are three different versions of Grafana. On the one hand, there is a free to download and locally installable package called Open-source version, where the user administers their own installation. The second available version is Grafana Cloud; in this case, Grafana Labs is responsible for administering and managing the service, including both free and paid options. The third option is the Grafana Enterprise, which imposes purchasing a license to access its premium features, being oriented to organizations with privacy and security requirements.

Creating a connection between visual panels and data sources is carried out by means of a simple editor interface, facilitating the creation of dashboards and reducing the deployment time. Alternatively, Grafana provides a Structured Query Language (SQL) editor that the developer uses to define the data to be queried and has advanced display options. Furthermore, embedding SQL code is another possibility for increasing the functionalities of this platform. For instance, mathematical equations can be coded through SQL in order to implement models that simulate the operation of physical devices using the data that are being queried and displayed [57].

The client user can easily access the Internet from devices like computers, tablets or smartphones using common web browsers, without the need of installing packages on the client side. Username and password credentials are required to connect to the dashboard. In particular, the user must indicate the Internet Protocol (IP) address and the logical port of the IoT server as well as their credentials (username and password) to establish a connection over the network.

The user is able to select the time interval of interest so that instant, short and long-term data can be easily displayed [58]. The ability to select different presentation intervals is a relevant feature for R&D projects [48]. In addition, this package offers numerous tools to deeply analyze the data such as tendency, median, mean and standard deviation [46]. Each panel can be displayed in full screen as well as zoomed to achieve detailed visualizations, fostering the supervision of the monitored process [58]. The visualized data can be locally downloaded enabling the analysis of such data in other software and allowing a wider access to data [47].

**Figure 1:**
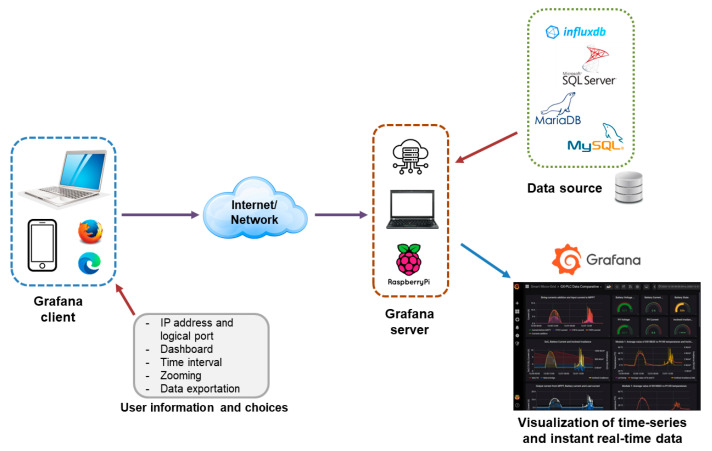
Block diagram of access to Grafana dashboards.

Figure 1 depicts a block diagram to illustrate the remote access from the user to the Grafana dashboard as well as the interactions with the user preferences and data sources. It must be noted that different devices are used to represent both the client and server sides in order to emphasize the versatility of this software.

Additionally, alerts generation is also a capability of this suite. When a variable reaches a certain threshold defined by the developer, an alert is created and shown in the dashboard. Indeed, instant notifications with such alerts can be sent through platforms like Slack or Telegram, enhancing the reaction of the operator to solve the anomalous situations.

Regarding users’ administration, Grafana provides a user and organization management interface that allows customized data access for users depending on the application [59]. Different permissions can be selected, creating users with edition capabilities or restricted to the visualization of the gathered data.

Moreover, continuous improvements are provided by means of plugins, which can be freely downloaded in most cases since they derive from the open-source IoT community on the Internet. These plugins enable additional functionalities such as visualization in the form of histograms, heatmaps, synoptics and webcam access, to name a few.

### 2.2. Brief Review of Grafana Applications

**Figure 2:**
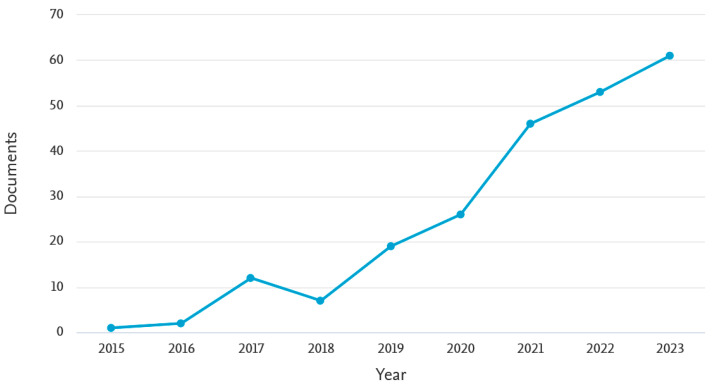
Publications dealing with Grafana over time according to the Scopus database.

The advantages of this software have led to its application in a number of facilities within different scientific scopes during the last decade. A search in the Scopus scientific database witnesses the number of publications that involve Grafana. Namely, from 2014 to December 2023, 227 publications have included Grafana. The evolution over time can be observed in Figure 2, showing an increasing trend. Moreover, 54 publications in the year 2024 have not been represented in order to achieve a clearer view of the publication trend. Indeed, it must be noted that there are many papers that use Grafana but do not include it in its metadata; therefore, the real amount is larger.

As a matter of fact, to aim at illustrating the applicability and versatility of this suite in scientific research, some recent publications in different scopes are discussed below.

In energy scope, this software has been profusely used. For instance, in [59], Grafana is applied to display the time series of temperature, humidity, solar radiation, etc., measured in a building monitored through an open-source proposal. The works [48,51] describe a PV-powered water pumping system where the user interface is developed with Grafana. A PV plant is supervised by means of Grafana in [52]. Grafana is applied to track the temperatures of PV modules integrated in a microgrid in [45]. The operation of a hydrogen generator is surveyed by means of Grafana in [34]. Data from digital and analog temperature sensors in a PV generator are visualized using Grafana in [60]. In [49], a smart energy meter using a Raspberry Pi and Grafana is built to measure and monitor energy consumption in computer devices. An IoT-based SCADA system for large-scale PV plants is described in [61], employing Prometheus for alerting tasks and Grafana for monitoring systems.

In other scopes, recent applications are also found. The work in [50] presents a monitoring system for volcanic fumaroles using Grafana for the visualization of the sensed magnitudes. Variables of robotic arms for pick and place tasks are displayed by means of Grafana in the work reported in [20]. An energy monitoring approach of an industrial asset is developed with Grafana in [62]. An industrial Big Data analytics architecture is proposed in [43], where Grafana performs data visualization. In [63], Grafana is used to monitor data in the context of digital twining. Data visualization for a machine tool and a 3D printer is achieved through Grafana in an IIoT-supported cloud manufacturing system presented in [64]. Electrical parameters of machines are monitored by means of a Raspberry Pi and Grafana in a microservices architecture for IIoT in [23]. A strawberry farm is monitored by means of Grafana in [46]. For air-quality monitoring, Grafana is applied in [53,65]. A similar application is found in [21] since Grafana is used to visualize data about environmental and air-quality variables. Aviation trajectory visualization and analysis are conducted using Grafana in [66]. The European Organization for Nuclear Research (CERN) uses Grafana for monitoring the computing infrastructure, for example, in the large hadron collider [47,67].

## 3. Applied IoT Architecture

In this section, the IIoT architecture applied in this work is described after contextualizing the functional architectures in the scopes of I4.0 and IIoT.

### 3.1. Overview of Architectures for I4.0 and IIoT

The first architecture to comment on is the widely known Automation pyramid. It was established in 1990 by the International Society of Automation (ISA)-95 standard and proposes hierarchical levels ranging from the industrial process itself to business management systems. This framework entails a hierarchical communication, horizontal connection among the components at the same level and a vertical communication with those of the immediately superior and inferior layers. The main drawback of the Automation pyramid is that data are exchanged between adjacent levels and the integration of multiple vendors is not supported [2]. Replacing or modifying existing automation systems and communication networks with the latest technologies becomes challenging [68].

Therefore, the pyramid is not adequate to accommodate I4.0 and IIoT technologies and decentralized and more complex architectures have been recently conceived [69]. Namely, three-dimensional architectures have been proposed for both paradigms. To begin with, the Reference Architecture Model Industrie 4.0 (RAMI 4.0) was developed in 2015 by the German Electrical and Electronic Manufacturers Association (ZVEI) in collaboration with the German government Industrie 4.0 initiative. This architecture consists of a three-dimensional map considering three axes (Layers, Lifecycle and value stream, and Hierarchy levels) for a structured description and tracking of an asset throughout its entire lifecycle. In this framework, the functions are distributed over the network and all participants can communicate with each other across hierarchical levels [70].

The second architecture to remark is the Industrial Internet Reference Architecture (IIRA), introduced in 2015 by the Industrial Internet Consortium (IIC) of the United States. The IIRA consists of a layered model that considers four different perspectives: business, usage, functional and application, which are represented in a three-dimensional manner. Similar architectures are the Industrial Value Chain Reference Architecture (IVRA) developed in Japan and the Intelligent Manufacturing System Architecture (IMSA) designed in China.

These architectures have very high degrees of complexity and abstractness, which make their application in real scenarios difficult. As stated in [7], reference architectures provide the basic layout guidelines for IIoT applications; however, it is difficult to address the arising challenges just by following such architectures due to problems derived from heterogeneous technologies and diverse industrial usage.

Concerning IoT architectures, these are based on a two-dimensional layered division, facilitating their comprehension and practical application. Hardware, software, communications and functionalities are distributed in a different number of layers. The most general IoT architecture is composed of three layers whilst there are proposals that consider up to eight levels, lacking single reference architecture [12].

### 3.2. Description of the IIoT Architecture

As previously discussed, there is not a standard number of layers for IoT (or IIoT) architectures. In this research, four levels have been applied. This configuration has been previously reported in the literature [6,10,34,71,72] and is considered adequate to organize the required equipment and functionalities.

**Figure 3:**
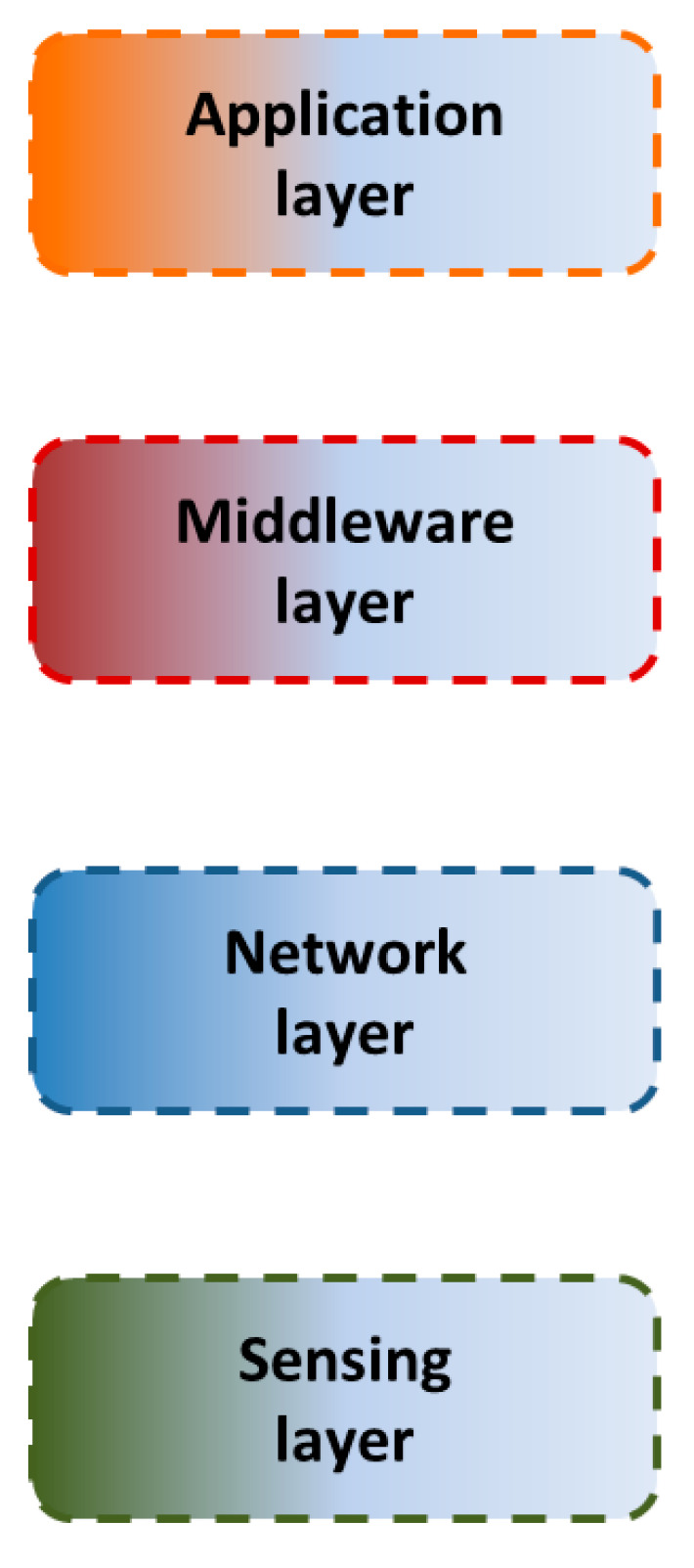
Block diagram of the IIoT architecture.

The layers that compose the applied architecture are described hereinafter and appreciated in Figure 3. Additionally, certain components used in the experimental cases are also discussed in the corresponding layer.

#### 3.2.1. Sensing Layer

This is a physical level that groups sensors and actuators devoted to exchange data from the real world, namely, the industrial process. Moreover, data acquisition and automation equipment also belong to this layer [10]. Therefore, the conversion from the analog world to digital information is carried out in this level in order to allow the rest of layers to manage the gathered data. In particular, in the present work, a Programmable Logic Controller (PLC) of the manufacturer Siemens (Munich, Germany, Europe) together with appropriate digital and analog Input/Output (I/O) modules are placed in this layer. Ethernet connectivity is achieved by embedded ports. This device provides reliable operation and modern communication interfaces, enabling its integration in an IIoT network. Indeed, this unit is representative of the automation resources that are commonly found in most industrial factories, in both new and legacy systems.

#### 3.2.2. Network Layer

The upper layer is the Network layer, which includes the technologies, communication protocols and interfaces for data transmission between the other layers [12]. Communication protocols commonly handled in this level are Transmission Control Protocol/Internet Protocol (TCP/IP), Wi-Fi, ZigBee, Constrained Application Protocol (CoAP), User Datagram Protocol (UDP) and Message Queuing Telemetry Transport (MQTT), to name a few. Network devices such as routers and switches as well as protocol converters are also included in this level.

Communication is a key enabler of IIoT [4], so this layer acquires special relevance for experimental scenarios. Indeed, as pointed out by Bader et al. [5], rather than a new design of future IIoT networks, the main target is managing the interoperability of heterogeneous systems. In fact, interoperability is a critical barrier to overcome for IIoT and I4.0 factories [25,38,44]. In particular—to deal with heterogeneity—in the present work, open and widely supported protocols are used. Namely, the version TCP of Modbus and the HyperText Transfer Protocol (HTTP) are applied. Modbus has a long trajectory in industry automation and the version TCP is profusely used in advanced industrial scenarios like the I4.0 and the IIoT [14,34,73]. Moreover, it is widely supported by proprietary and IoT open-source hardware and software. On the other hand, HTTP is considered a reliable communication protocol in the I4.0 and IIoT context [74], and it is used by the software Grafana to retrieve data from databases.

#### 3.2.3. Middleware Layer

The third layer, the Middleware layer, can be considered the core of the IoT system since it contains different components to perform operations required to develop IoT-based applications [75]. This layer is devoted to data collection and processing, including storage, filtering, aggregation, calculations and analysis of information from the Network layer. Data are retrieved from heterogeneous equipment through different communication protocols, providing abstraction from specific technologies. In fact, this middleware level enables interoperability among connected devices [76]. Furthermore, the combination of open communication protocols and middleware facilitates connectivity with legacy devices [69].

In the present research, two open-source and free middleware are considered. Namely, the programming language Python and the block-oriented Node-RED are used. It must be noted that both environments are being applied in a number of research approaches related to I4.0 and IIoT to handle data transmission [14,33]. For each of the experimental cases reported in the next chapter, specific details of the middleware will be provided.

Regarding data storage, the use of relational SQL databases is a trend in industrial systems [33] where the amount of information to be managed is very high [2]. To this aim, in the present architecture, an in-house cloud database implemented with the open-source software MariaDB is applied.

In contrast to timeseries databases, which use a structure associated with a timestamp such as InfluxDB or Prometheus, relational databases use a structure based on rows and columns. This general structure gives versatility to this database, allowing it to be implemented in a wide variety of applications.

Within an industrial environment, the use of databases such as MariaDB allows for the storage of non-time series information. As an example related to cybersecurity, these databases allow for the storage of information related to user profiles, passwords and permissions. This information can be used to verify the information entered by a user during the login process in a specific environment.

#### 3.2.4. Application Layer

The Application layer contains the user applications, such as web applications, and uses the components of the Middleware layer [75]. This top level provides output formats, applications and services requested by the final users [76]. In the industrial environment, software solutions and applications for remote monitoring and control are developed within this layer, including the visualization of the process evolution over time by means of graphical interfaces [10]. Hence, user interfaces enable the interaction between the user and the process, which are paramount when managing complex industrial systems [77]. Indeed, the recent literature highlights their role in Industry 4.0/5.0 and IIoT arenas [69,77,78].

Depending on the particular application scenario, different hardware and software entities will be used to fulfil the specific requirements in this top layer. Nonetheless, the present work includes Grafana for graphic representation in the Application layer regardless of the scenario given the advantages and capabilities that such software provides, as it has been described in the Section 2. This way, the database of the Middleware layer is used to feed the dashboards of Grafana where data are plotted and displayed to the final user in numerical and time-series formats.

## 4. Experimental Validation and Discussion

The suitability of the IIoT architecture is proven through experimental validation using real-world equipment, both hardware and software, of proprietary and open-source nature, and managing data under real operation conditions. It is important to emphasize that the results obtained from this experimental validation have been analyzed from a functional perspective, ensuring the proper operation and implementation of the IIoT architecture.

### 4.1. Microgrid Description

**Figure 4:**
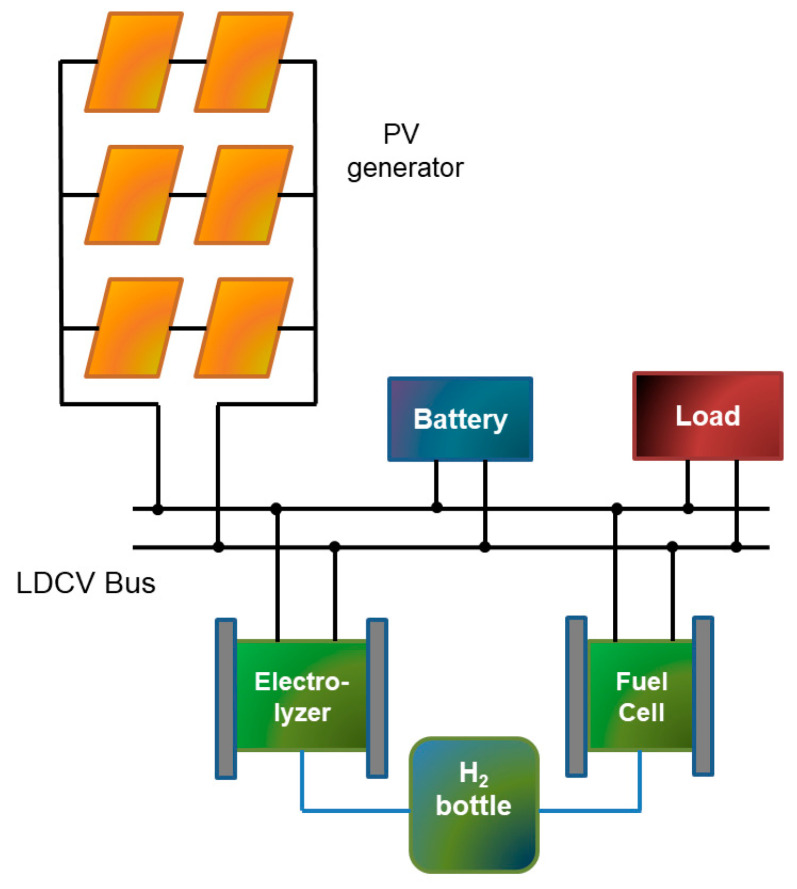
Schematic diagram of the microgrid used as the experimental application scenario.

A microgrid based on renewable energies and hydrogen has been used as the experimental facility to apply the IIoT architecture. This intelligent power grid combines distributed renewable generation and consumption with isolated autonomous operation. Namely, a photovoltaic (PV) generator produces electricity from the incident solar irradiance. This generation serves to feed a programmable electronic load as well as to supply a Lithium-ion battery. Such a battery acts as a low DC voltage (LDCV) bus around which the power flows take place. Green hydrogen is produced by dispatching PV power to a hydrogen generator in order to take advantage of available power when the battery is fully charged, and the load is also supplied. Hydrogen is accumulated in metal-hydride bottles, which constitute the hydrogen bus. In the opposite situation, the stored hydrogen is converted into electricity through a fuel cell. Figure 4 illustrates schematically the microgrid.

Apart from the research about energy aspects, this facility requires data acquisition, transmission, storage and visualization, involving different hardware and software entities. Therefore, the microgrid has served as a benchmark to develop and test different architectures and monitoring systems under the frameworks of the I4.0 and the IIoT. In particular, four subsystems have been considered and treated as experimental scenarios separately to deal with the heterogeneity of the aforementioned different hardware and software. This way, the IIoT architecture expounded in the previous section has been applied to the four subsystems taking into account the particularities and involved equipment, demonstrating the suitability of such architecture. The application of the architecture is described for each subsystem in the next subsections.

### 4.2. PV Generator

**Figure 5:**
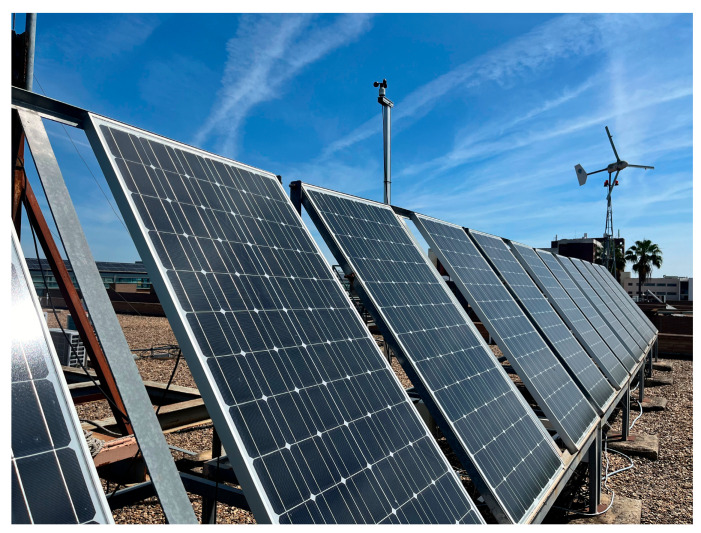
Physical aspect of the PV modules that compose the generator.

The PV generator is composed of six monocrystalline modules of 185 W and 24 V of nominal voltage, providing a total power of 1.1 kW. The photograph of the PV generator can be appreciated in Figure 5. Such a generator is placed on the rooftop of the building along which the microgrid equipment is distributed.

The operation of the PV generator is characterized by the following magnitudes: temperature, current, voltage and incident irradiance. Consequently, a set of sensors have been mounted and connected to measure these variables. The temperature is sensed by a Pt-100 probe placed in the back plane of each module. The voltage is measured by means of potentiometric voltage dividers whilst the current is sensed using Hall effect sensors. A current sensor is used for each string of the generator. Regarding the solar irradiance, a pyranometer in the same plane of the PV modules is used. In addition, the ambient temperature is also sensed through a Pt-100 probe.

A Remote Input/Output Station (RIOS) placed close to the PV generator is used to connect the sensors and to send the measurements to the PLC through the fieldbus PROFINET. Table 1 summarizes the signals and sensors used for this purpose.

**Figure 6:**
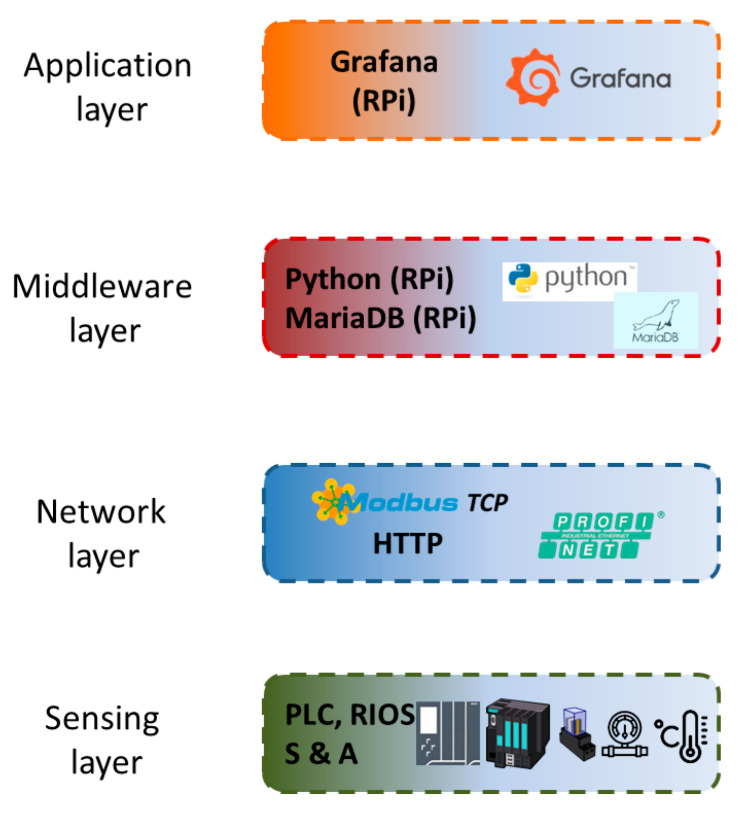
Block diagram of the IIoT architecture applied to monitor the PV generator.

Figure 6 depicts the IIoT architecture particularized to monitor the described PV generator. As it can be observed, the lower layer comprises the aforesaid sensors, the PLC and the RIOS. The measured data are transmitted through PROFINET, Modbus TCP and HTTP, and these protocols are grouped in the Network layer. The Middleware layer includes two software components, MariaDB for data storage and Python for data sharing. In particular, a Python script is used to establish Modbus TCP and SQL connections, so the data sensed in the PV generator are read from the PLC and written in the database, respectively. Lastly, the Grafana suite is placed in the top level, the Application layer, where a GUI has been deployed to display the most relevant magnitudes in the form of time-series and instant values. The plotted information is read by Grafana through HTTP from the database and the user accesses it by means of a web browser. Concerning hardware equipment, both Middleware and Application layers are hosted by a SBC, namely a Raspberry Pi.

**Figure 7:**
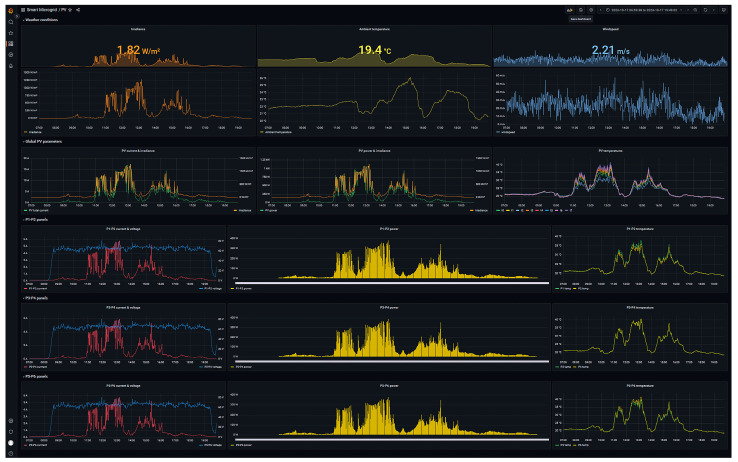
Monitor interface for the PV generator.

Aiming at illustrating the achieved results, the designed monitoring interface is shown in Figure 7 and discussed. The interface is structured in five rows that collect the various graphical elements in the following groups:

- Weather conditions: where incident solar irradiance, ambient temperature and wind speed are represented. These parameters are illustrated by means of instant numerical values as well as graphical time trends.
- Global parameters: where the electrical generation (in current and power) is visualized together with the solar irradiance, as well as the variation in the temperature of the PV panels.
- P1–P2 panels: whose graphical elements represent the evolution of the current, voltage, generated power and temperature of the panels comprising the first string.
- P3–P4 panels: homologous to the previous row and dedicated to second string.
- P5–P6 panels: homologous to the previous row and dedicated to third string.

On the one hand, instant values allow for a rapid inspection of the current state of the generator working conditions, namely, solar irradiance, ambient temperature and wind speed. On the other hand, time-series charts are devoted to displaying the historical trend of the magnitudes, facilitating the observation of the operation evolution as well as trends. The developer is able to group different variables in the charts in order to design a representative graph of the facility, in this case, the PV generator. In this sense, the total current generated by the three strings is depicted together with the irradiance. Furthermore, the combined representation of the temperatures of the panels is highlighted.

**Figure 8:**
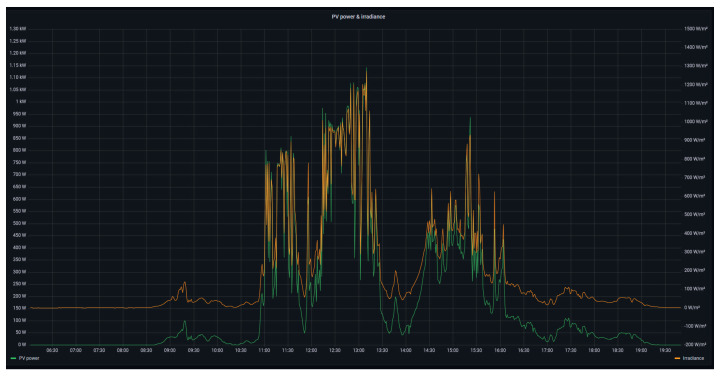
PV-generated power and solar irradiance.

As a detail of the operation of the PV generator, Figure 8 illustrates the generated power (left axis, green curve) together with the solar irradiance (right axis, orange curve). As shown in the figure, the generated power describes a parallel curve to the irradiance due to the direct dependence of both parameters. The offset between the represented curves has been configured in order to observe in detail the simultaneous evolution of both variables, thus avoiding a total overlapping along the representation.

### 4.3. Battery

**Figure 9:**
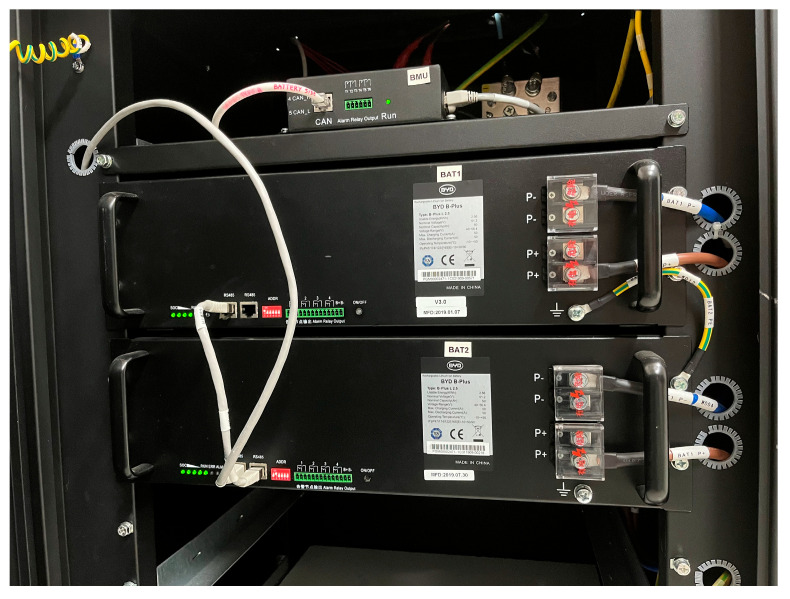
Physical aspect of the battery modules.

The Lithium-ion battery acts as an electrochemical energy storage means and as a DC bus of the microgrid. It is an iron phosphate battery composed of two modules of 2.5 kW each, electrically connected in parallel, model B-Box Pro 5.0, manufactured by BYD. The battery is equipped with an embedded electronic unit (Battery Management Unit, BMU) responsible for measuring the variables of the battery (voltage, current and temperature) as well as to estimate its State of Charge (SoC) and State of Health (SoH). This unit is provided by the manufacturer and cannot be accessed or modified by the user. In Figure 9, the aspect of both modules alongside the BMU (top position) can be observed.

The BMU is completely closed and without any configuration option; therefore, to access the measured and calculated data hosted by this device, a gateway of a different manufacturer is required. In this case, the chosen device is the Colour Control GX of Victron Energy. This device collects the data of the battery operation through the Controller Area Network (CAN) bus and makes this information accessible to third party equipment by means of MQTT or Modbus TCP protocols.

However, apart from this gateway, for a higher generality, the architecture includes a PLC that has connected current and voltage sensors for direct measurement of both magnitudes regardless of the BMU of the manufacturer. Moreover, the current delivered to the electronic load is also measured by the PLC. The battery temperature, the SoC and the SoH (which cannot be measured) are exchanged with the gateway. Table 2 summarizes the signals and sensors used for this purpose. It must be remarked that the manufacturer of the battery does not provide information about its sensors, so in the table the source device (the gateway) is indicated instead of the sensor model.

**Figure 10:**
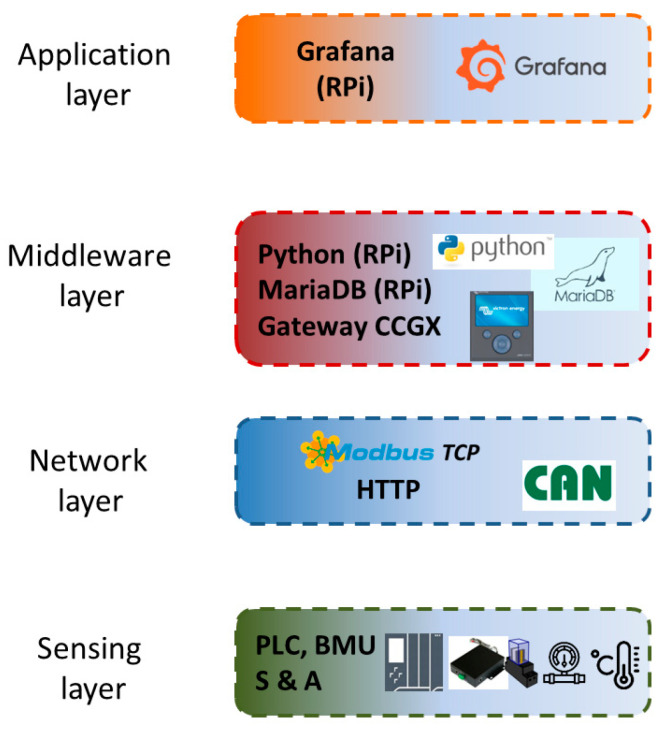
Block diagram of the IIoT architecture applied to monitor the battery.

The IIoT architecture for this case is represented in Figure 10. The Sensing layer groups the sensors, the PLC and the BMU. The Network layer hosts the protocols CAN, Modbus TCP and HTTP. The components of the Middleware layer are Python, MariaDB and the gateway CCGX. Both the PLC and the gateway act as Modbus servers, whereas a Python script plays the role of Modbus client. This way, the Python code acquires the battery representative magnitudes and sends them to the database through SQL statements. Real-time graphic visualization of the battery operation is achieved by means of a Grafana interface, materializing the Application layer. Concerning hardware, the software components of the Application and the Middleware layers run in Raspberry Pi-based SBC, with the exception of the aforesaid gateway.

**Figure 11:**
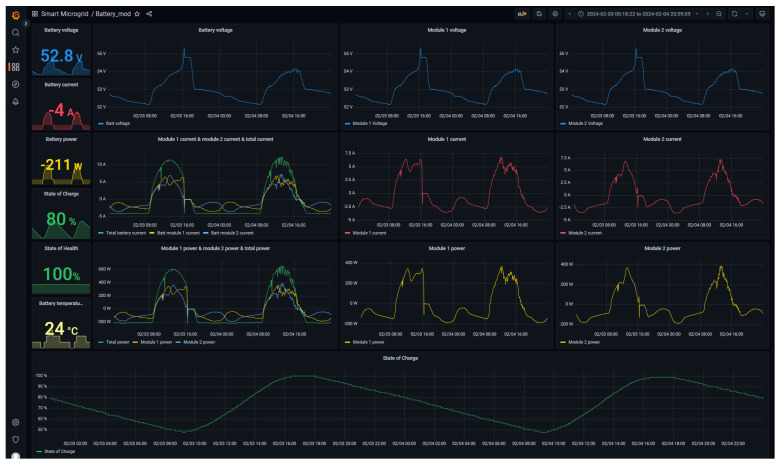
Monitor interface for battery.

The designed monitoring interface is shown in Figure 11 and discussed. The graphic elements that integrate the interface have been arranged in columns according to the following distribution:

- Instant values: this column illustrates the instant values of the key parameters of the battery by means of numerical indicators; namely, the battery voltage, current, power, SoC, SoH and temperature are depicted.
- Global parameters and comparison: in this column there are three trend graphs representing the evolution of the battery voltage, the total current and the current of each module, as well as the total power together with the power of each module.
- Module 1: this module displays the values associated with the voltage, current and power of the first battery module using trend graphs.
- Module 2: this module is homologous to the previous column and dedicated to the second module.

Lastly, the evolution of the battery SoC is displayed at the lower part of the interface screen.

**Figure 12:**
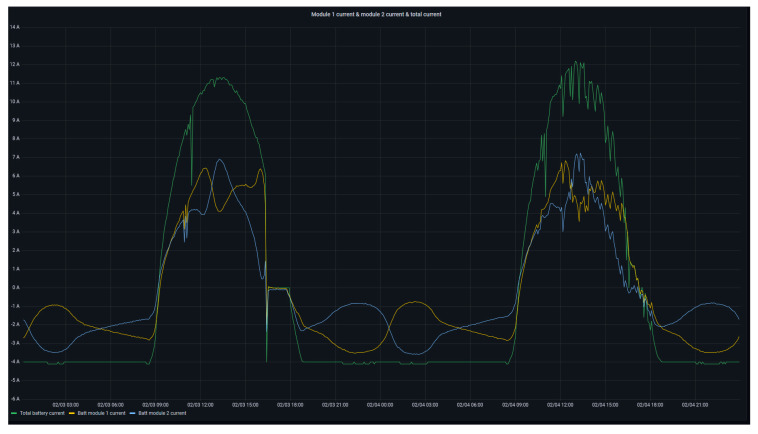
Total battery and modules current.

Figure 12 presents in more detail the variation of the total current of the battery (green curve) as well as the current of module 1 (yellow curve) and module 2 (blue curve). This graph depicts the operation of the battery under constant load consumption. During the evolution of the variables, the events of charging and discharging of the battery are differentiated as a consequence of a surplus and deficit of energy production, respectively. During the discharge event (night hours), the operation of the BMU is highlighted, whose management balances the current supplied by each module in order to equalize the individual SoC of each of them.

### 4.4. Hydrogen Generator

**Figure 13:**
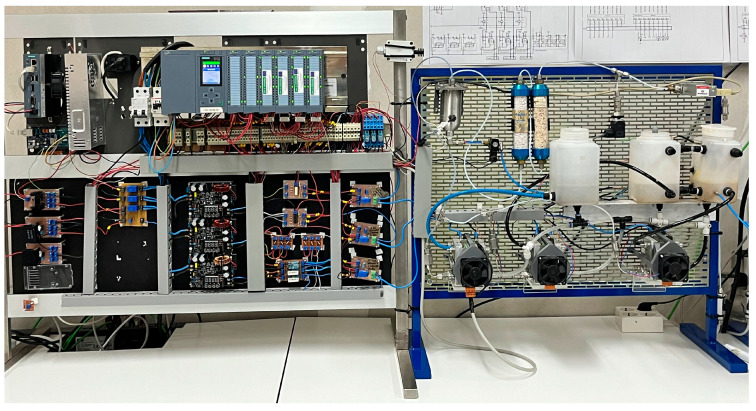
Physical aspect of the hydrogen generator.

The operation of a hydrogen generator, also called electrolyzer, is based on an installation consisting of three polymer electrolyte membrane (PEM) stacks that constitute the third practical case. In this installation, carrying out the water electrolysis process, the hydrogen flow that can be produced is 750 mL/min and the consumed current can reach up to 10 A. A photograph of experimental setup is seen in Figure 13. In this facility, sensors and actuators are needed to manage the electrochemical process, namely, the input current, the stack voltage and temperature, and the produced hydrogen flow and pressure are measured. Refer to [34] for further details. Table 3 summarizes the signals and sensors used for this purpose.

**Figure 14:**
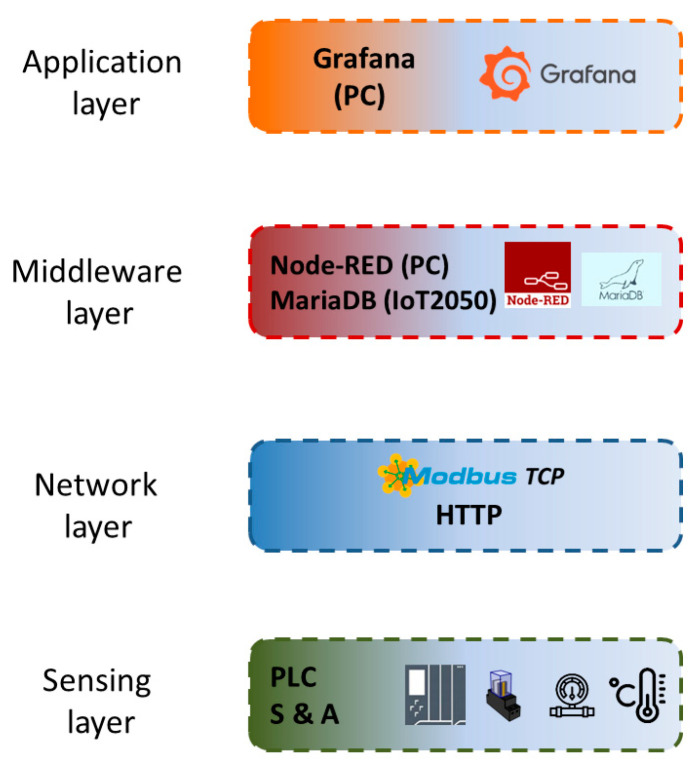
Block diagram of the IIoT architecture applied to monitor the hydrogen generator.

The four layers of the IIoT architecture are depicted in Figure 14 for the hydrogen generator. The previously discussed sensors and actuators alongside the PLC to which they are connected constitute the Sensing level. The Network layer involves the protocols Modbus TCP and HTTP. In the Middleware layer, MariaDB and Node-RED are found. In this case, a Modbus TCP linkage is established between Node-RED and the PLC to fetch the process signals. For data accumulation, SQL code is programmed in a JavaScript function node for writing such signals. Data visualization is achieved through a Grafana interface that displays the magnitudes hosted in the database. The Grafana server and Node-RED run in an MS Windows PC, whereas in the Middleware layer, the computation device to host the database is an IIoT gateway manufactured by Siemens, the model IoT2050. Despite being commercial equipment, it is of an open-source nature, providing great configuration capabilities to the developer.

**Figure 15:**
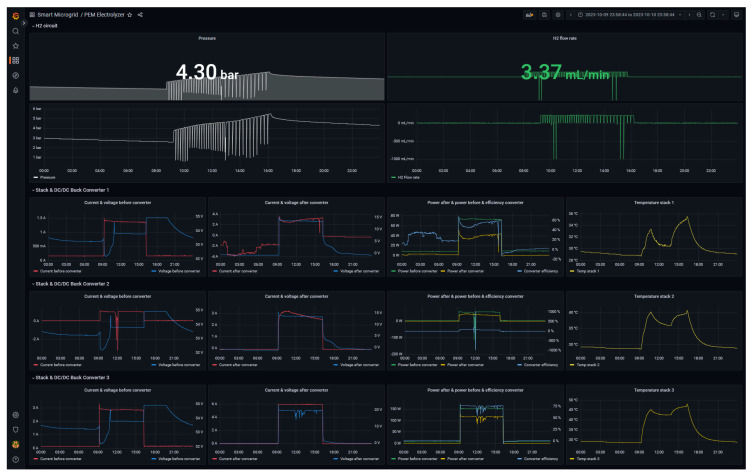
Monitor interface for the hydrogen generator.

The designed GUI is shown in Figure 15 and discussed. The interface is structured in four rows that collect the various graphical elements in the following groups:

- H_2_ circuit: this row illustrates the instant value and the historical trend of the working pressure and the generated hydrogen flow rate.
- Stack & DC/DC Buck Converter 1: this group is focused on the first stack and collects the evolution of current, voltage and power at the input and output of the converter. Furthermore, this group displays the efficiency of the converter as well as the working temperature of the stack.
- Stack & DC/DC Buck Converter 2: homologous to the previous row and dedicated to stack and converter 2.
- Stack & DC/DC Buck Converter 3: homologous to the previous row and dedicated to stack and converter 3.

**Figure 16:**
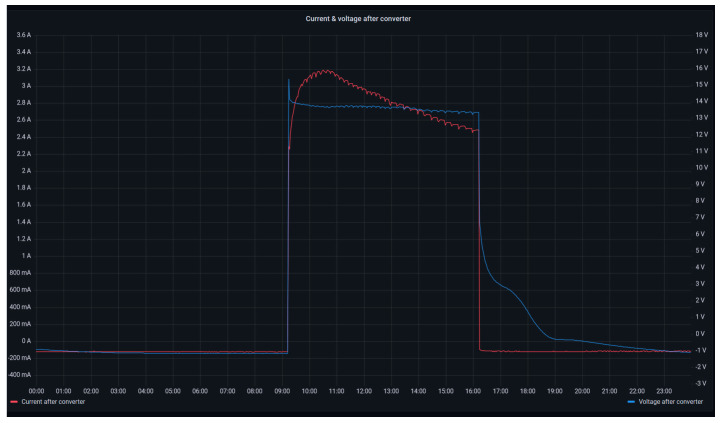
Stack 2 voltage and current.

Figure 16 illustrates the operation of stack 2 by means of the evolution of the consumed current (left axis, red curve) and the stack voltage (right axis, blue curve). This graph depicts the rapid response of the PEM stack, where the voltage tracking upon a sudden increase of the consumed current (stack start-up) is highlighted. Furthermore, the robustness of the voltage during the nominal operation of the device is emphasized. Lastly, in the stack shutdown process, the self-discharge effect of the PEM cells is visualized through the progressive decrease of the stack voltage curve [79].

### 4.5. Fuel Cell

**Figure 17:**
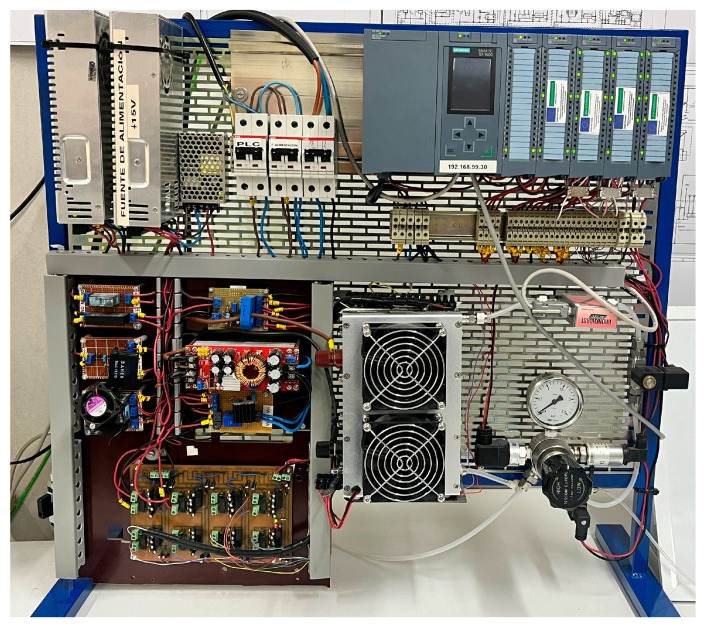
Physical aspect of the hydrogen fuel cell.

The last application corresponds to sensing and monitoring the hydrogen consumption to generate electricity. Namely, a hydrogen PEM fuel cell model Horizon 500 is the core device in this experimental case. It provides a maximum power of 0.5 kW and is fed from the hydrogen bus to supply the load when there is not enough PV generation. Its physical appearance can be seen in Figure 17.

Similar to previous equipment, this device is equipped with a set of sensors and actuators connected to a PLC for handling its operation, materializing the Sensing Layer. Input signals are the flow and pressure of the consumed hydrogen, the produced current and voltage as well as the temperature of the stack. Table 4 summarizes the signals and sensors used for this purpose.

**Figure 18:**
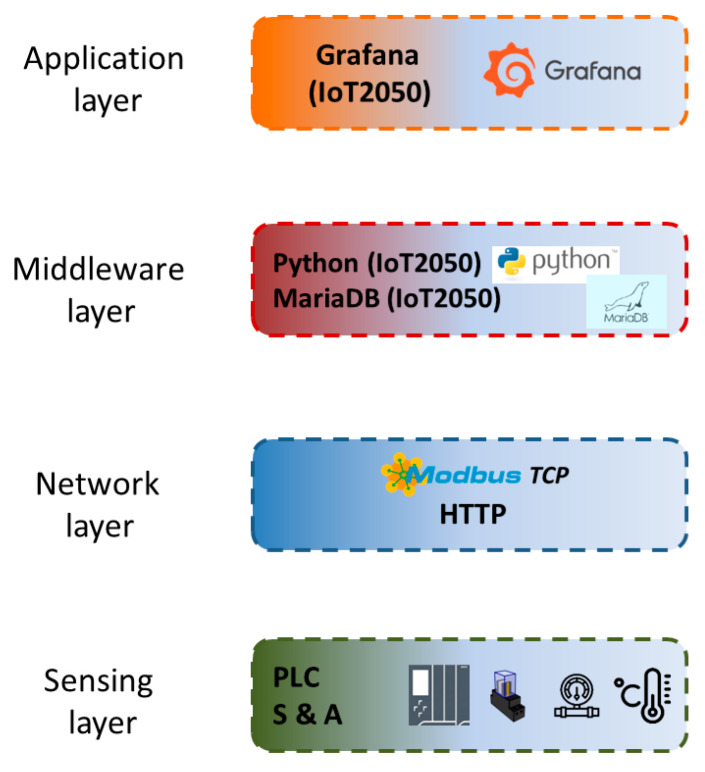
Block diagram of the IIoT architecture applied to monitor the fuel cell.

As can be seen in Figure 18, the sensing layer includes, apart from the aforesaid sensors, the industrial PLC to which they are connected. The Network layer covers the protocols HTTP and Modbus TCP. Regarding the third layer, a Python script performs the middleware functions, retrieving data from the PLC through Modbus TCP and sending the information to a database implemented with MariaDB. The Grafana-based interface, as well as in previous cases, displays such information in the form of time-series charts and instant values for a proper visualization of the fuel cell status. In this application case, the Grafana server, the MariaDB database and the Python script run in an industrial gateway manufactured by Siemens, model IoT2050.

**Figure 19:**
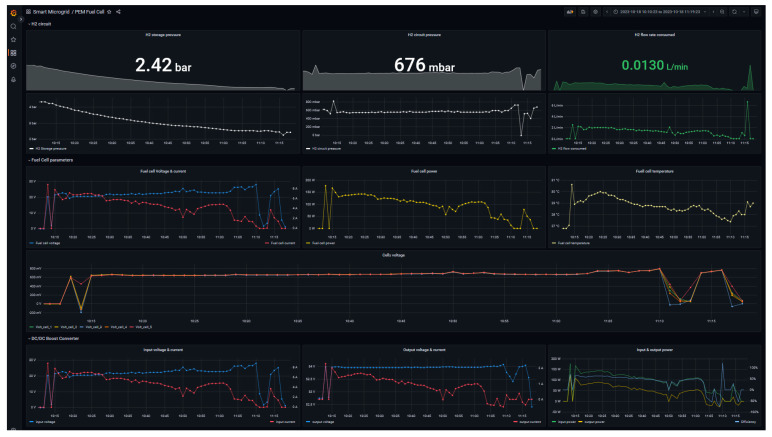
Monitor interface for fuel cell.

The designed GUI is shown in Figure 19 and discussed. The interface is structured in three rows that collect the various graphical elements in the following groups:

- H_2_ circuit: this row illustrates the instant value and the historical trend of the pressure of H_2_ storage system, pressure of H_2_ circuit and H_2_ flow rate consumed.
- Fuel cell parameters: this space is dedicated to the representation of the key parameters of the fuel cell such as total voltage and current, generated power, working temperature as well as the variations of individual cells voltage.
- DC/DC Boost Converter: this last row is composed of a graph plotting the variables of the DC/DC boost converter, namely, input voltage and current, output voltage and current, and input power and output power together with efficiency are depicted.

**Figure 20:**
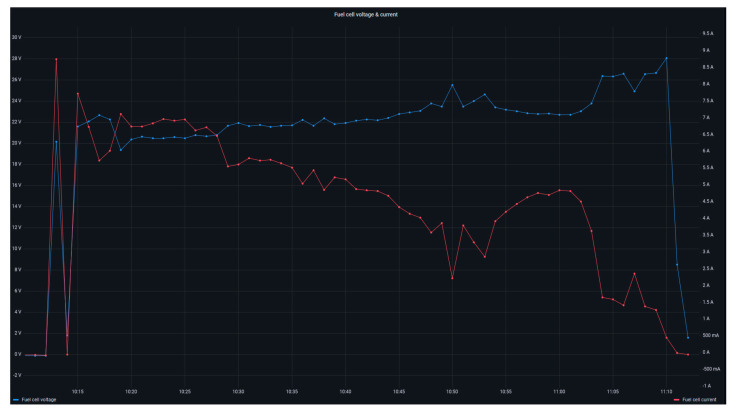
Fuel cell current and voltage.

Figure 20 displays the operation of the fuel cell by means of the evolution of voltage (left axis, blue curve) together with the generated current (right axis, red curve). Similar to the electrolyzer, the cells that comprise the fuel cell are PEM type; thus, the rapid response characteristic of this cell architecture can be observed in the graph. This response is noticeable in the start-up and shutdown sections, where the voltage instantly follows the variations in the current setpoint.

On a general basis for the four applications described, a data acquisition and storage time of 1 min has been set. This period is considered sufficient to monitor the response and operation of the equipment involved. However, the flexibility of the Middleware layer (Python/Node-RED–MariaDB) allows the developer to adjust this parameter if a shorter period is required.

Regarding the volume of data stored, Table 5 shows the total number of variables measured, the sampling period used and the total number of records in the database. Each record contains the values of all the variables measured for a specific time instant.

Furthermore, the proper operation of the Grafana environment through the visualization of the stored data facilitates the verification of the operation of the rest of the layers of the architecture, ensuring the following:

- The hardware elements of the Sensing layer send their information correctly to the PLC.
- The elements of the Middleware layer perform the reading and storage of the installation data by means of the protocols established at the Network layer.
- The Grafana environment (Application layer) communicates correctly with the MariaDB database and allows the reading and visualization of the stored data.

In order to summarize the exposed cases, Table 6 portrays the components for each layer of the architecture in the four experimentation cases. It must be noted that for simplification in the table, only the most illustrative sensors of the Sensing layer are shown, since the rest of the sensors and actuators have been described in previous subsections.

### 4.6. Discussion

The validity of the architecture has been successfully proven through experimental application to several different subsystems involving a number of hardware and software nodes as well as operation under real conditions.

The Grafana-based monitoring system is platform independent and can run on low-cost hardware, avoiding the dependence of MS Windows of common SCADA solutions [33]. In particular, different hardware nodes have been used to deploy the Grafana server taking advantage of its multi-platform and lightweight features. It was executed successfully in MS Windows PC and in Linux-based SBC (Raspberry Pi and IoT2050).

Regarding the Middleware layer, two different suites were applied, Node-RED and Python. For the PV generator, battery, and fuel cell subsystems, a custom Python code was employed. Although this code was adjusted for each subsystem, its internal structure was similar; first, communication was established with the PLC via the Modbus TCP protocol. Next, the data contained in the PLC were read. Finally, the data were stored by means of an SQL query to the MariaDB database. This common structure facilitated the extension of the code and thus the scalability of the proposed IIoT architecture in the Python environment. For the hydrogen generator subsystem, a similar structure Node-RED flow was deployed. In both cases, the task of data sharing through different protocols was properly addressed. Nonetheless, the visual flow-based framework and easy interaction features of Node-RED facilitated such a task, reducing time and efforts.

Still, in the Middleware layer, the use of gateways with IoT capabilities is a necessity in modern facilities to allow data access. In the presented cases, both open-source and commercial devices were integrated to facilitate data sharing. It must be noted that the importance of this type of equipment is highlighted in the literature for IIoT facilities [64].

MariaDB was used in all the reported cases providing easy usage and free cost. This feature is aligned with the trend of monitoring/supervisory systems towards connecting to SQL databases [33]. Indeed, given the compatibility of Grafana with other database management systems, different databases can be used without requiring significant modifications in the architecture.

Regarding communications, it must be noted that the Modbus TCP protocol is supported by industrial proprietary and IoT open-source equipment, which makes it a proper communication protocol to address the standardization and interoperability issues in I4.0 and IIoT facilities [34,58,80].

Using IoT open-source software for monitoring purposes provides an important reduction of expenses devoted to SCADA systems. The complexity and cost of conventional monitoring methods have recently increased the importance of IoT-based monitoring systems [81]. With open-source suites like Grafana, licensing and upgrading costs can be avoided, which could reach thousands of dollars/euros if proprietary supervisory/monitoring software was preferred [30,33].

The use and integration of both proprietary and open-source hardware equipment was successful. The importance of open-source tools has been emphasized in the previous literature for IIoT and I4.0 [30,31,32], as well as for the energy sector [37]. In this regard, the required software for every layer has been of an IoT open-source nature. An exception is found in the case of configuring and programming the automation units, such as PLC, since commercial software was required. Namely, the TIA Portal of the manufacturer Siemens with the associated licensing costs was used.

PLCs are the prevalent automation technology in industrial environments and are also considered an essential part of the I4.0 and IIoT frameworks [42,82]. In the reported experimental cases, this component was used to connect sensors and actuators as well as needed to implement energy management strategies. Nonetheless, if required, for the application of the architecture, this equipment can be replaced by IoT devices dedicated to acquiring data from sensors.

This work has reported concrete details of hardware, software and communications in order to contribute to the advancement of IIoT and I4.0 facilities avoiding the abstractness and lack of definition in architectures found in much of the literature [3,7,11].

Some limitations of the work are commented on as follows. Firstly, a boundary of Grafana is that it cannot exchange data directly with the memory of a PLC, which could be interesting for an industrial context. However, the heterogeneity of equipment (hardware, software, communications and vendors) commonly imposes the inclusion of middleware to handle such heterogeneity. Middleware can be in the form of software, like Node-RED for example, or hardware, like gateways; therefore, Grafana is capable of managing sensors and PLC data through the intermediation of middleware.

Further research guidelines are oriented towards including other modern communication protocols like MQTT in the Network layer. This protocol is the standard for IoT ecosystems and is supported by much equipment. In recent years, industrial equipment like PLC and SCADA systems are progressively supporting this protocol [2]. Moreover, developing a fifth layer, the so-called Business layer, to implement digital twins of the hydrogen generation and consumption equipment of the microgrid will be approached, taking advantage of the presented architecture. Furthermore, adding cybersecurity measures will also be considered in future developments given the importance of this aspect in modern I4.0 and IIoT-enabled facilities [6,28,35].

Regarding these cybersecurity issues, the software environments involved in the proposed IIoT architecture present the following typologies of vulnerabilities, where some recent Common Vulnerabilities and Exposures (CVE) are highlighted:

- Inherent vulnerabilities in the software version, which are solved and lead to the release of a new version of the program. Some examples include the cases associated with the MariaDB (CVE-2024-21096) and Grafana (CVE-2024-9476) environments.
- Vulnerabilities due to functions associated with packages/plugins. The use of outdated packages increases the risk of attacks due to the exploitation of vulnerabilities. The most frequent attacks in these cases are Denial of Service (DoS) attacks. As an example of this typology, a vulnerability in Node-RED associated with the Node.js “cross-spawn” package has recently been fixed (CVE-2024-21538).
- Vulnerabilities associated with SQL code injection. Environments such as MariaDB and Grafana work through requests formed in SQL code. Malicious access to these environments facilitates the attacker to execute malicious SQL code, allowing the inclusion, modification and elimination of information, thus altering the information contained in the database and the operation of the environments.

## 5. Conclusions

IIoT developments are deployed according to functional-layered architectures. There is a large variety of architecture proposals in the scientific literature mainly under conceptual designs. However, their experimental application has been scarcely reported, many times centered on particular aspects.

Aiming at overcoming these limitations, this paper has presented the experimental application of an IIoT architecture divided into four functional layers, namely, Sensing, Network, Middleware and Application layers. A pilot microgrid has served as a scenario to test and prove the suitability of the architecture in four application cases. The validation of the architecture has been performed using automation hardware (PLC) from a well-known manufacturer (Siemens) alongside IoT software (Grafana, MariaDB, Node-Red, Python) with increasing presence in industrial factories. IoT communication protocols such as Modbus TCP and HTTP, among others, have enabled seamless data transmission.

Special focus has been put on the IoT software Grafana for visualization in the top level of the architecture, the so-called Application layer. The most relevant magnitudes of the experimental equipment have been displayed using time-series graphs. This software, which is gaining attention from scientists and engineers, allows for the development of user interfaces with great compatibility with different databases and operative systems, as well as easy-to-use interaction through web navigation.

The target groups are scientists, engineers and practitioners in the scope of IIoT, who can find useful insights and practical implementation cases. This paper is expected to act as a useful resource and to encourage the real application of IIoT technologies.

## Figures and Tables

**Table 1:** PV generator signals and sensors.

Signal	Sensor
Temperature	Pt-100
Voltage	Potentiometric voltage divider
Current	Hall effect sensor
Irradiance	Pyranometer

**Table 2:** Battery signals and sensors/data source.

Signal	Sensor
Current	Hall effect sensor
Voltage	Potentiometric voltage divider
SoC	Gateway
SoH	Gateway
Temperature	Gateway

**Table 3:** Hydrogen generator signals and sensors.

Signal	Sensor
Current	Hall effect sensor
Voltage	Potentiometric voltage divider
Temperature	Pt-100
Pressure	Pressure transmitter
Hydrogen flow	Thermal mass flow meter

**Table 4:** Signals and sensors for the fuel cell.

Signal	Sensor
Current	Hall effect sensor
Voltage	Potentiometric voltage divider
Temperature	Pt-100
Pressure	Pressure transmitter
Hydrogen flow	Thermal mass flow meter

**Table 5:** Data storage for each subsystem.

Hardware	Variables	Sample Period	Total Number of Records
PV generator	16	1 min	1.625.319
Battery	12	1 min	1.625.319
Hydrogen generator	26	1 min	347.329
Fuel cell	19	1 min	521.674

**Table 6:** Hardware, software and communications for each layer in each experimental case.

Layer	PV Generator	Battery	Hydrogen Generator	Fuel Cell
Application layer	Grafana	Grafana	Grafana	Grafana
	Raspberry Pi	Raspberry Pi	PC	IoT2050
Middleware layer	Python, MariaDB	Python, MariaDB	Node-RED, MariaDB	Python, MariaDB
	Raspberry Pi	Raspberry Pi, Gateway	PC, IoT2050	IoT2050
Network layer	Modbus TCP, HTTP, PROFINET	Modbus TCP, HTTP, CAN	Modbus TCP, HTTP	Modbus TCP, HTTP
Sensing layer	PLC, RIOS	PLC, BMU	PLC	PLC
	Temperature, irradiance, voltage, current	Temperature, voltage, current, SoC	Temperature, hydrogen flow, voltage, current	Temperature, hydrogen flow, voltage, current

## Data Availability

The datasets presented in this article are not readily available because the data are part of an ongoing study. Requests to access the datasets should be directed to authors.
